# Comprehensive analyses of molecular features, prognostic values, and regulatory functionalities of m^6^A-modified long non-coding RNAs in lung adenocarcinoma

**DOI:** 10.1186/s13148-023-01475-z

**Published:** 2023-04-07

**Authors:** Yili Ping, Jingjuan Huang, Jichao Zhu, Zujun Sun, Anquan Shang, Chen Chen, Wenfang Liu, Dong Li

**Affiliations:** 1 Department of Laboratory Medicine, Shanghai Tongji Hospital, Tongji University, School of Medicine, Shanghai, 200065 China; 2 Department of Laboratory Medicine, Zhejiang Provincial Hospital of Chinese Medicine, Hangzhou, 310000 China; 3 Department of Laboratory Medicine, Huzhou Central Hospital, Huzhou, 313099 China; 4 Department of General Surgery, Shanghai Tongji Hospital, Tongji University, School of Medicine, Shanghai, 200065 China

**Keywords:** Lung adenocarcinoma, N6-methyladenosine, LncRNA, Prognostic values, Bioinformatics analysis

## Abstract

**Background:**

Lung adenocarcinoma (LUAD) has a high incidence and recurrence rate. N6-methyladenosine (m^6^A) modification of RNA has become a promising epigenetic marker in tumors. The dysregulation of both RNA m^6^A levels and m^6^A regulator expression levels reportedly affects essential biological processes in various tumors. Long non-coding RNAs (lncRNAs), a subgroup of RNAs over 200 nucleotides in length that do not code for protein, can be modified and regulated by m^6^A, but the relevant profile in LUAD remains unclear.

**Results:**

The m^6^A levels of total RNA were decreased in LUAD tumor tissues and cells. Multiple m^6^A regulators were abnormally expressed at both the RNA and protein levels, and were related in expression patterns and functionally synergistic. Our microarray revealed 2846 m^6^A-modified lncRNA transcripts as well as its molecular features, 143 of which were differentially m^6^A-modified and manifested a negative correlation between expression levels and m^6^A modification levels. More than half of the differentially m^6^A-modified lncRNAs associated with dysregulated expression. The 6-MRlncRNA risk signature was a reliable indicator for assessing survival time of LUAD patients. The competitive endogenous regulatory network suggested a potential m^6^A-induced pathogenicity in LUAD.

**Conclusions:**

These data have demonstrated that differential RNA m^6^A modification and m^6^A regulator expression levels were identified in LUAD patients. In addition, this study provides evidence increasing the understanding of molecular features, prognostic values, and regulatory functionalities of m^6^A-modified lncRNAs in LUAD.

**Supplementary Information:**

The online version contains supplementary material available at 10.1186/s13148-023-01475-z.

## Background

According to GLOBOCAN 2020 statistics, lung cancer was the second most common tumor type and had the highest mortality worldwide [1]. Despite the great improvements in antitumor treatments over the past three decades, the 5-year survival rates for patients with regional and widespread disseminated lung cancer were only 27% and 4%, respectively [2]. Notably, as the most prevalent lung cancer histological subtype, lung adenocarcinoma (LUAD) accounted for about 40% of cases [3] and has had increasing morbidity in recent years [4], making it the main research focus in this field. To improve the therapeutic effect and prolong patient survival, novel effective biomarkers are required for the early detection, prognosis, and monitoring of LUAD, as well as to explore the molecular mechanisms underlying this disease.

N6-methyladenosine (m^6^A), the most prevalent post-transcriptional modification in human mRNAs, was discovered within the consensus motifs DRm^6^ACH (D = G/A/U, R = G/A, H = A/U/C) and is highly enriched within long internal exons, around stop codons, and in the 3′ untranslated region (3′ UTR) of mRNAs [5, 6]. Such cellular modifications are part of a highly dynamic state that is regulated by three types of RNA m^6^A regulators, including methyltransferases (METTL3, METTL14, METTL16, VIRMA, RBM15, RBM15B, WTAP, ZC3H13, and CBLL1), demethylases (FTO and ALKBH5), and binding functional proteins (YTHDF1/2/3, YTHDC1/2, HNRNPC/A2B1, RBMX, IGF2BP1/2/3, FMR1, EIF3A, ABCF1, ELAVL1, G3BP1/2, ZNF217, and LRPPRC). These regulator types mediate the occurrence, removal, and functionality of m^6^A modifications, respectively [7–10]. Several studies have found that an abnormal abundance of RNA m^6^A modifications, likely mediated by the mutation or aberrant expression of m^6^A regulators, is a new epigenetic gene regulatory mechanism in cell differentiation and proliferation in certain tumors [11, 12].

Long non-coding RNAs (lncRNAs), defined as a type of RNA molecule that is more than 200 nucleotides in length and has limited or non-existent protein coding ability, are poorly conserved among species and show dynamic and specific expression patterns in various tissues and cells [13, 14]. Because lncRNAs were found to be generally far lower as abundant as mRNAs in a wide range of human organs, they were initially believed to be a kind of transcriptional byproduct [15, 16]. However, researchers later demonstrated that lncRNAs can significantly impact cell proliferation and differentiation in tumor cells because of their multiple roles in transcriptional and post-transcriptional regulation of certain oncogenes [17]. Currently, the m^6^A modification was found in many lncRNAs and could support their functionality in regulating the expression levels of tumor-related genes via affecting self-stability [18], nuclear accumulation [19], RNA–protein interactions [20], and affinity of binding microRNAs (miRNAs) [21]. Nevertheless, the m^6^A-modified lncRNA profile in LUAD remains unclear.

In this study, we first evaluated and confirmed the relationship between the m^6^A modification and LUAD by detecting the m^6^A modification levels in total RNA and exploring the m^6^A regulator expression levels. Importantly, the Arraystar Human M^6^A-modified LncRNA Epitranscriptomic Microarray was utilized to analyze the characteristics of m^6^A-modified lncRNAs and screen out lncRNAs with differential methylation level in LUAD. Combined analysis with The Cancer Genome Atlas (TCGA) database, we developed and validated the six m^6^A-regulated lncRNAs (6-MRlncRNA) risk signature to predict the overall survival (OS) of LUAD patients. At last, we predicted the competitive endogenous regulatory role of differentially m^6^A-modified lncRNAs in LUAD.

## Results

### LUAD is associated with low levels of total m^6^A-modified RNA

**Fig. 1:**
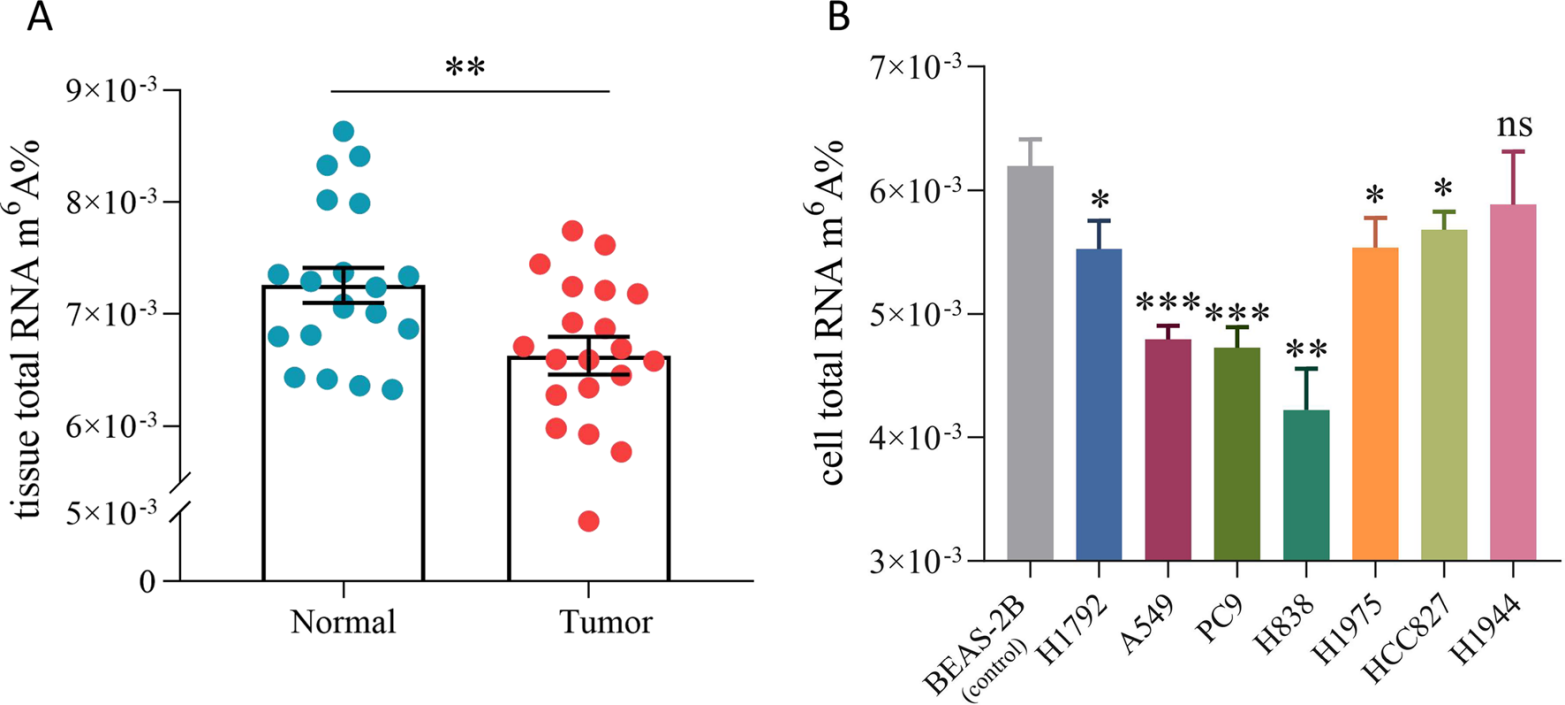
The m^6^A levels of total RNA from LUAD tissues and cell lines. **A** The m^6^A level of total RNA in twenty paired LUAD patients’ tumor tissues (Tumor) and matched adjacent non-tumor tissues (Normal). The bar represented mean ± SEM. Paired t test. **P < 0.01. **B** The m^6^A level of total RNA in LUAD cell lines and a normal epithelial cell line (BEAS-2B). Unpaired t test. *P < 0.05, **P < 0.01, ***P < 0.001, not significant (ns) *P* > 0.05

To explore the potential role of m^6^A modification in LUAD, we first examined the m^6^A levels in total RNA samples. We found that the majority of early-stage LUAD tumor tissues exhibited reduced RNA m^6^A methylation levels compared with adjacent non-tumor lung tissues (*N* = 20, *P* < 0.01) using the EpiQuik™ m^6^A RNA methylation quantification kit (Fig. 1A). Consistently, the m^6^A modification abundance of RNA from the six LUAD cell lines was lower (*P* < 0.05) compared with that from the normal lung epithelial cell BEAS-2B. This was particularly clear in the H838, PC9, and A549 cell lines (Fig. 1B). These results indicated that the RNA m^6^A modification level was decreased in LUAD tissues and cells.

### Disordered expression of m^6^A regulators in LUAD tissues and cell lines

**Fig. 2:**
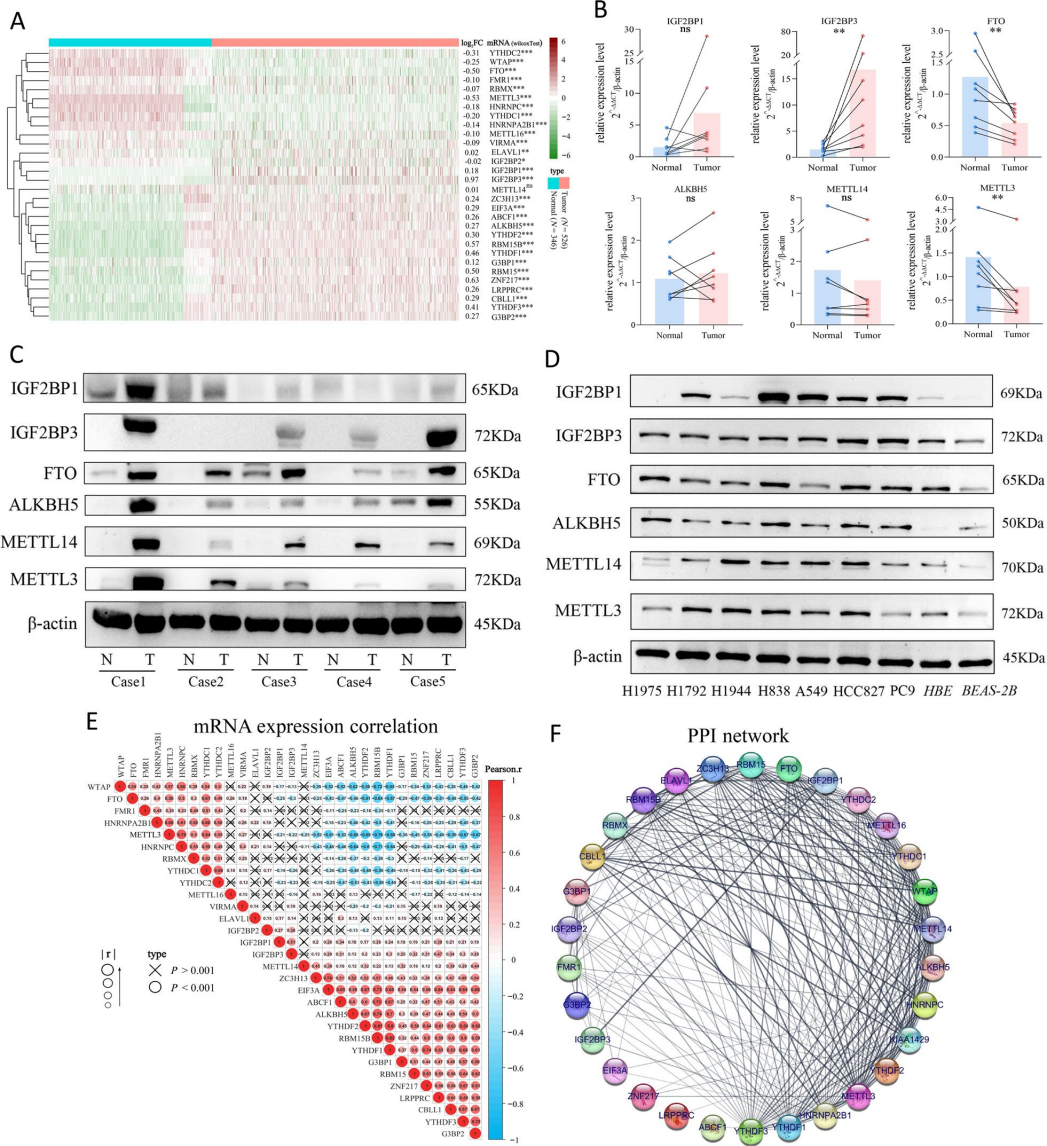
The expression levels of m^6^A regulators in LUAD and the correlation between m^6^A regulators. **A** Heatmap of the expression levels of m^6^A regulators (LUAD tumor tissues vs. normal lung tissues) in TCGA and GTEx databases. FC represented the expression fold change of T-median/N-median. WilcoxTest. *P < 0.05, **P < 0.01, ***P < 0.001 and not significant (ns) *P* > 0.05. **B** RT-qPCR showed the relative mRNA expression levels of METTL3, METTL14, ALKBH5, FTO, and IGF2BP1/3 in eight paired LUAD tissues (Tumor) and adjacent non-tumor tissues (Normal). Paired t test. **P < 0.01 and not significant (ns) *P* > 0.05. **C, D** Western blots showed the protein expression of METTL3, METTL14, FTO, ALKBH5, and IGF2BP1/3 in LUAD tissues (**C**) and cell lines (**D**), respectively. **E** The Pearson correlation analysis of m^6^A regulators’ expression levels in 872 samples in TCGA and GTEx databases. The number in the circle represented the Pearson coefficient, which determined the size of circle. The cross in the circle represented no correlation (*P* > 0.001). **F** The PPI network analysis of m^6^A regulators. Line thickness indicated the strength of data support

We hypothesized that RNA m^6^A modification could be more broadly associated with the altered expression levels of m^6^A methyltransferases, demethylases, and functional proteins in LUAD. Thus, we evaluated the gene expression levels of 30 reported m^6^A regulators. Through the conjoint analysis of TCGA and GTEx databases, the transcript expression levels of these regulators were all disordered in LUAD tumor tissues (all *P* < 0.05, except for METTL14), but all differences were not significant (all |log_2_FC|< 1) (Fig. 2A). Verification by RT-qPCR indicated that METTL3 and FTO were decreased (*P* < 0.01), and IGF2BP3 was increased (*P* < 0.01) in LUAD. METTL14, ALKBH5, and IGF2BP1 didn’t show a significant statistical difference (Fig. 2B). Overall, the disordered expression patterns of six regulators in eight paired clinical LUAD samples showed similar trends as the database analysis results. Next, western blot analysis revealed that the six regulators’ protein levels were all significantly increased in LUAD tumor tissues (Fig. 2C), which were consistent with the results in LUAD cell lines with BEAS-2B or HBE cells as the normal control (Fig. 2D). Only IGF2BP3 showed coincident differences in transcript and protein levels, both of which were elevated in LUAD. These results suggested that the disordered expression patterns of m^6^A regulators were likely involved in the development of LUAD.

To further exploring the potential relationship of the 30 m^6^A regulators, we investigated their RNA expression relationship and protein interaction by Pearson coefficient and PPI network analysis, respectively. Beyond METTL16, most m^6^A regulators had correlations with at least one other regulator at the transcript level in lung tissues (|*r*|> 0.3, *P* < 0.001), and the strongest negative and positive co-expressions were METTL3 and RBM15B (*r* = − 0.76, *P* < 0.001), and YTHDF1 and RBM15B (*r* = 0.82, *P* < 0.001) (Fig. 2E). Additionally, most of m^6^A regulators had frequent protein interactions with each other, especially with WTAP, METTL14, ALKBH5, and HNRNPC (Fig. 2F), suggesting a cooperative interaction pattern in LUAD.

### Overview of m^6^A-modified LncRNA microarray in LUAD tissues

**Fig. 3:**
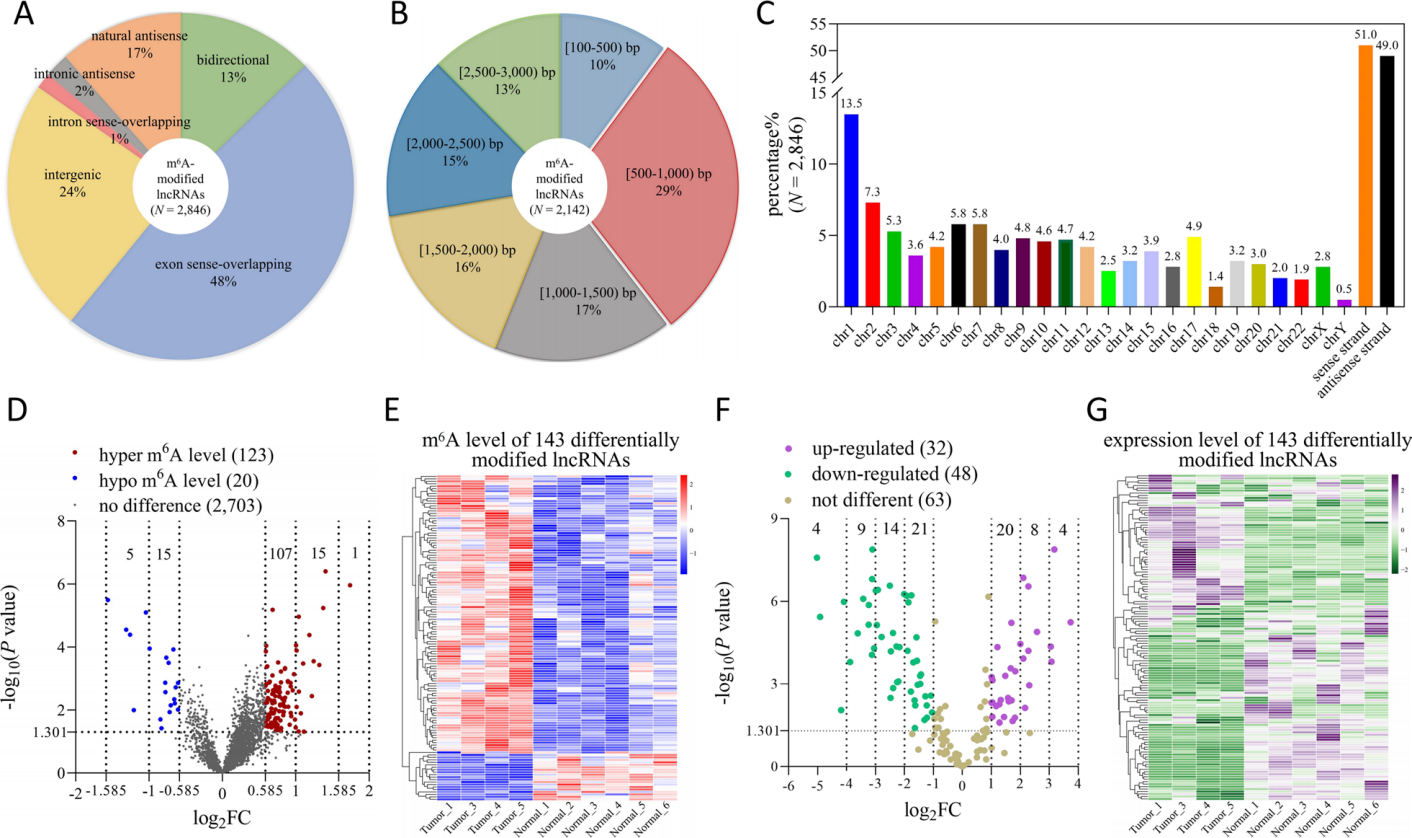
Characteristics of the m^6^A-modified lncRNA microarray in LUAD tumor tissues and adjacent normal tissues. **A** The source distribution of m^6^A-modified lncRNA transcripts. **B** The length distribution of m^6^A-modified lncRNA transcripts (≤ 3000 bp). **C** The chromosomal localization of m^6^A-modified lncRNA transcripts. **D** Volcano plot showed that in 2846 m^6^A-modified lncRNA transcripts, 143 were differentially modified (|log_2_FC|> 0.585, *P* < 0.05, unpaired t test), and 123 were hypermethylated and 20 were hypomethylated. **E** The methylation level heatmap of 143 differentially modified lncRNA transcripts. **F** Volcano plot showed that in 143 differentially modified lncRNA transcripts, 32 were upregulated and 48 were downregulated (|log_2_FC|> 1, *P* < 0.05, unpaired t test). **G** The expression level heatmap of 143 differentially modified lncRNA transcripts

The above experiments demonstrated that m^6^A regulator expression levels were abnormal in LUAD, making us speculate that lncRNAs modulated by these m^6^A regulators may also have characteristics changes. To examine the m^6^A-modified lncRNA profile, we performed the Arraystar Human m^6^A-lncRNA Epitranscriptomic Microarray on six paired LUAD tumor tissues and adjacent normal tissues. In 12,496 lncRNA-specific probes, the microarray hybridized to and identified a total of 2846 m^6^A-modified lncRNA transcripts, whose source distribution were mainly in exon sense-overlapping (48%), intergenic (24%), natural antisense (17%), and bidirectional (13%) (Fig. 3A). The length distributions of 2142 m^6^A-modified lncRNA transcripts (≤ 3000 bp) were mostly 500 to 1000 bp (29%), while there was no significant proportion difference in the other length ranges, which were all 10% to 17% (Fig. 3B). The m^6^A-modified lncRNA transcripts were localized to all chromosomes, with chromosomes 1 and Y having the highest (13.5%) and lowest (0.5%) abundance, respectively (Fig. 3C). In addition, the amounts of transcripts originating from sense and antisense strands had no apparent difference (Fig. 3C).

To improve biological repetition and eliminate intra-group error, we more specifically examined the data of samples with great heterogeneity (Pearson.*r* < 0.9) according to the sample correlation analysis of lncRNA expression levels before statistical analysis (Additional file 1: Fig. S1). Finally, the data of Tumor_2/6 were omitted, and the data of other four tumor tissues (Tumor_1/3/4/5) and six adjacent normal tissues (Normal_1/2/3/4/5/6) were used for follow-up difference analysis. Collectively, 123 hypermethylated and 20 hypomethylated lncRNA transcripts had different m^6^A methylation levels in LUAD tumor tissues (|log_2_FC|> 0.585, *P* < 0.05) (Fig. 3D and Additional file 2: Table S1), which were displayed in the clustering heatmap (Fig. 3E). We observed that most changes in m^6^A methylation levels were within a twofold difference. Subsequently, the expression analysis of 143 differentially modified lncRNA transcripts indicated that 32 were upregulated and 48 were downregulated (|log_2_FC|> 1, *P* < 0.05) (Fig. 3F, G and Additional file 2: Table S2). These expression differences ranged from twofold to 37-fold, much higher than the observed fold changes for the m^6^A modification level. The additional 63 differentially modified lncRNA transcripts did not show significantly differential expression levels (|log_2_FC|< 1 or *P* > 0.05) (Fig. 3F, G and Additional file 2: Table S2).

### Combined analysis of the m^6^A modification profile and expression profile in LUAD tissues

**Fig. 4:**
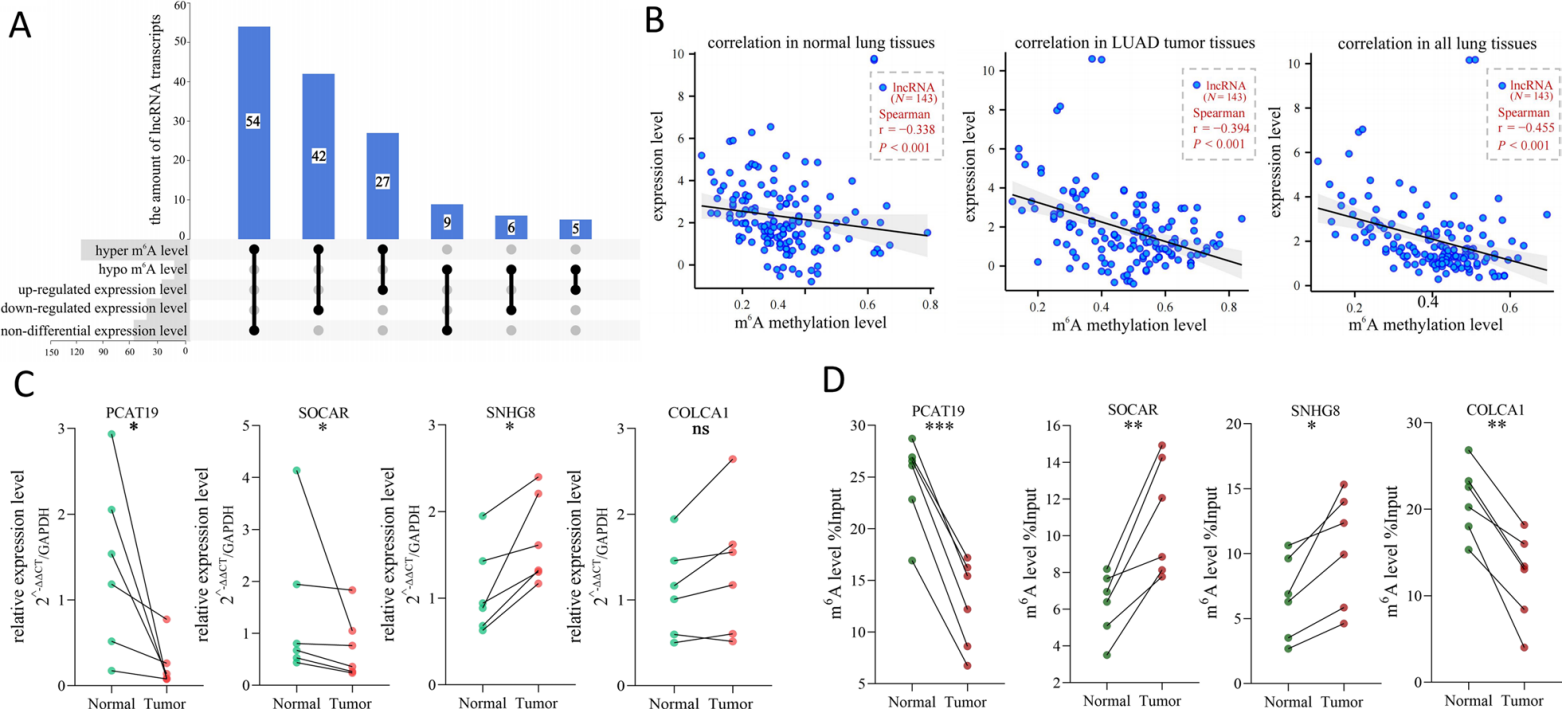
Combined analyses of m^6^A modification and expression profiles of 143 differentially m^6^A-modified lncRNA transcripts in LUAD tissues. **A** The UpSet diagram was used to divide lncRNAs into six categories. **B** Spearman correlation analyses between the expression levels and methylation levels of 143 lncRNA transcripts in normal tissues (left), tumor tissues (middle), and all tissues (right). A point’s horizontal and vertical coordinates were the mean m^6^A level and mean expression level of a lncRNA transcript in six normal tissues (left), four tumor tissues (middle), and all ten tissues (right), respectively. **C** Validation of four lncRNAs’ expression levels in six pairs of clinical tissues; GAPDH served as the reference gene. *P < 0.05 and not significant (ns) *P* > 0.05. Paired t test. **D** Validation of four lncRNAs’ m^6^A levels in six pairs of clinical tissues by MeRIP-qPCR assays; Input was used as the reference. *P < 0.05, **P < 0.01 and ***P < 0.001. Paired t test

Because epigenetic modifications often affect expression levels of the gene, we speculated that the difference in lncRNA expression levels may be caused by its altered m^6^A modification level. To investigate these, the UpSet diagram was exploited to divide the 143 differentially modified lncRNA transcripts into six categories on the basis of the alteration of both modification and expression (Fig. 4A and Additional file 2: Table S3). We found that “hyper–not different” was the most numerous category (*N* = 54), followed by “hyper–down” (*N* = 42), “hyper–up” (*N* = 27), “hypo–not different” (*N* = 9), “hypo–down” (*N* = 6) and “hypo–up” (*N* = 5). Next, we performed Spearman correlation analysis between the expression levels and m^6^A levels of the 143 lncRNA transcripts in normal tissues, tumor tissues, and both in normal and tumor tissues, respectively. The results showed a negative relationship, suggesting that in LUAD, a differentially m^6^A-modified lncRNA with a higher m^6^A methylation level may have a lower expression level (Fig. 4B). Subsequently, four differentially m^6^A-modified lncRNAs, SOCAR, PCAT19, SNHG8 and COLCA1, were selected to evaluate the accuracy of the microarray result. As shown in Fig. 4C, RT-qPCR on clinical tissue samples revealed enhanced expression of SNHG8, decreased expression of SOCAR and PCAT19, and unaltered expression of COLCA1 in LUAD. Additionally, MeRIP-qPCR assays validated that SOCAR and SNHG8 transcripts were hypermethylated, and PCAT19 and COLCA1 transcripts were hypomethylated in LUAD tumor tissues (Fig. 4D). The experimental validation results of SNHG8 and SOCAR were consistent with the results in the microarray, which indicated that the microarray had accuracy and its analyses had reference significance. Overall, these results suggested that disordered expression patterns of lncRNA associated with abnormally altered m^6^A modification on lncRNA.

### Construction and evaluation of the m^6^A-regulated LncRNA prognostic signature

**Fig. 5:**
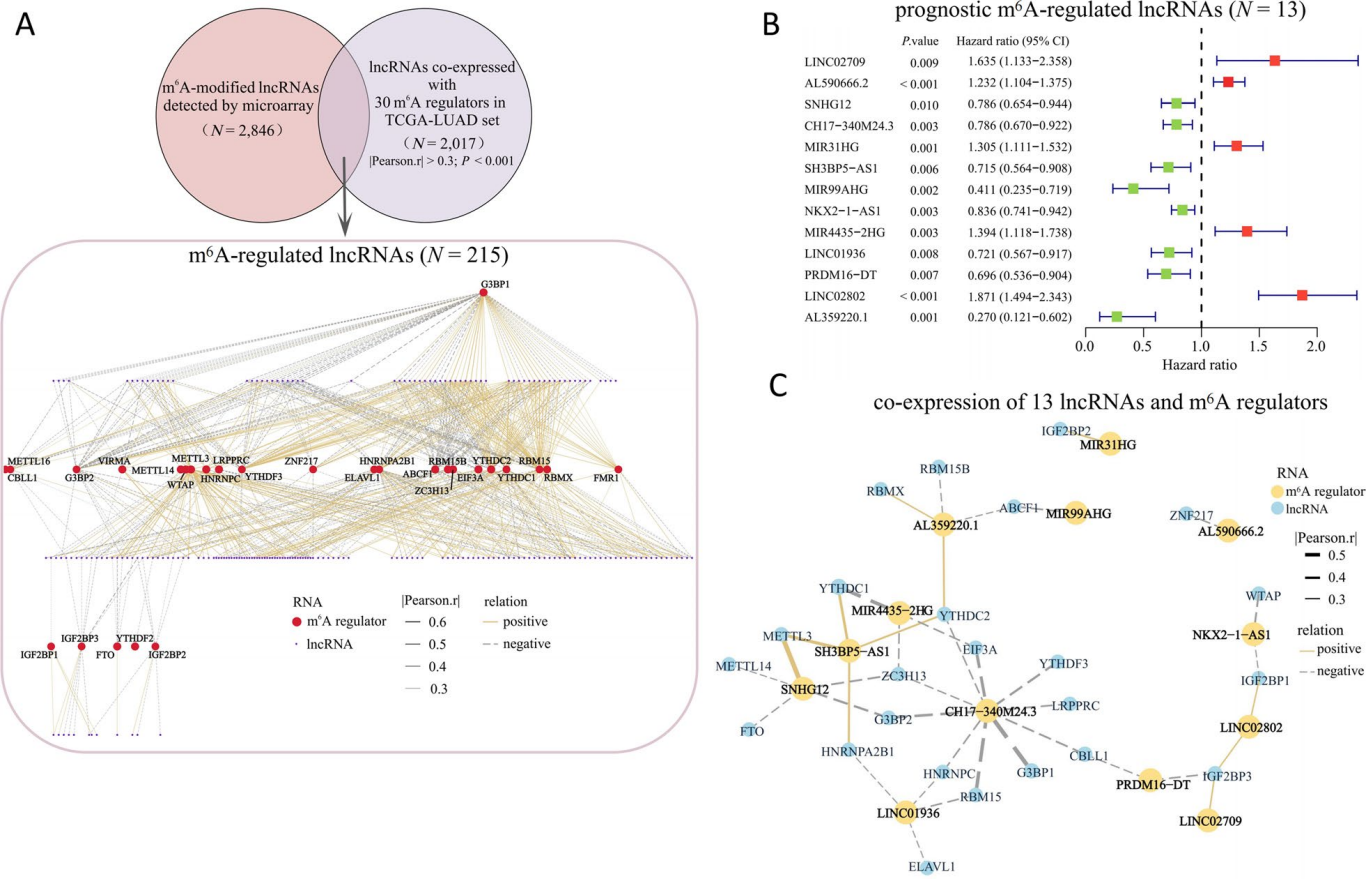
The screening of prognostic m^6^A-regulated lncRNAs. **A** The screening criteria of 215 m^6^A-regulated lncRNAs and its expression correlation with m^6^A regulators. **B** Forest plot of univariate Cox regression analysis of m^6^A-regulated lncRNAs (*P* < 0.01). The hazard ratio (HR) value, its 95% confidence interval (CI), as well as the associated *P* value, were shown. HR > 1 indicated that the lncRNA was a risk factor and its high expression was unfavorable for prognosis, while the high expression of the protective lncRNA of HR < 1 was favorable for prognosis. **C** The expression correlation between 13 prognostic m^6^A-regulated lncRNAs and m^6^A regulators

**Fig. 6:**
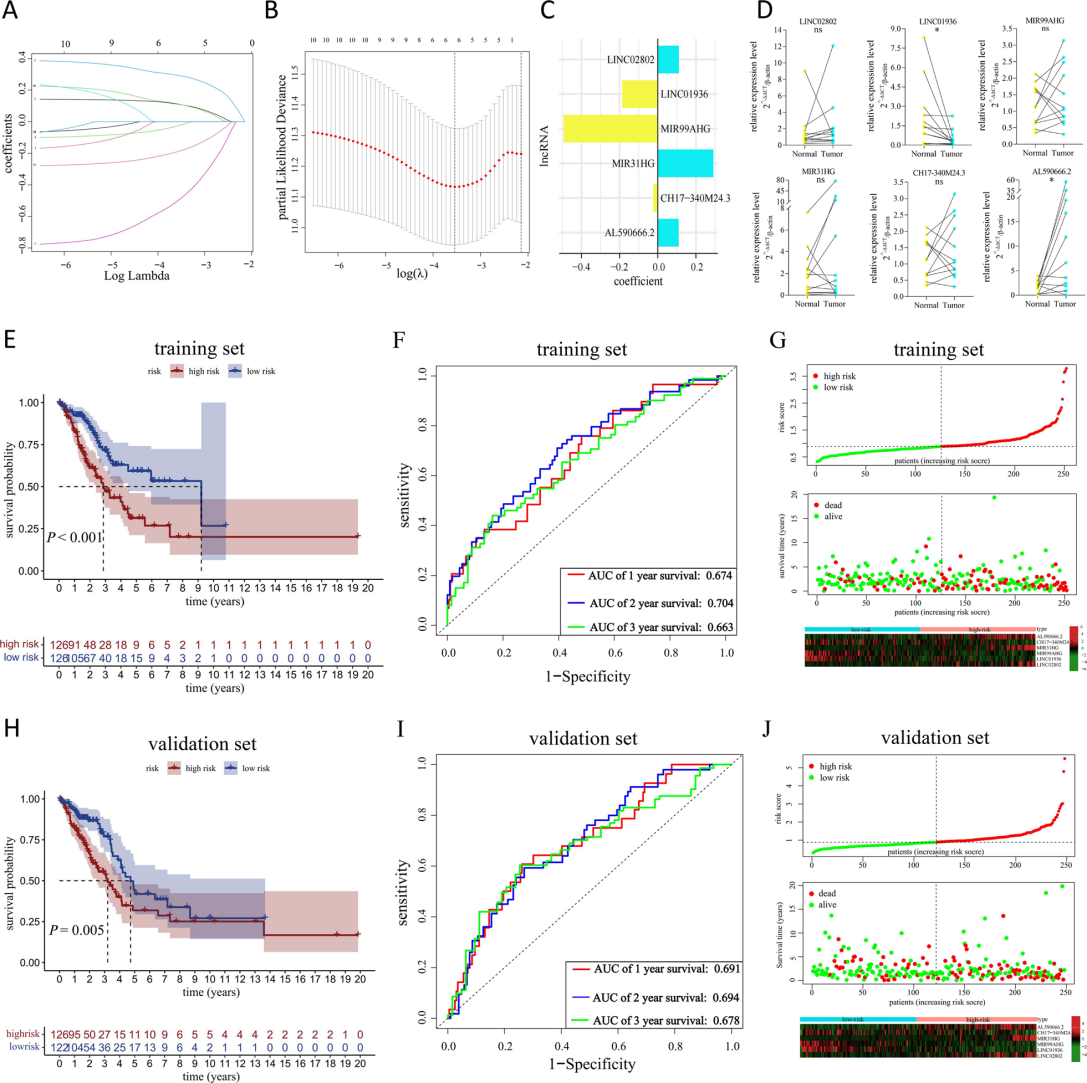
Construction and validation of the 6-MRlncRNA prognostic signature in LUAD. **A, B** The Lasso coefficient values and vertical dashed lines were calculated at the best log (lambda) value. **C** Lasso coefficient profiles of six prognostic m^6^A-regulated lncRNAs in the signature were displayed. **D** RT-qPCR was used to examine the relative expression levels of the six m^6^A-regulated lncRNAs in 12 paired LUAD tissues (Tumor) and adjacent non-tumor tissues (Normal). *P < 0.05, not significant (ns) *P* > 0.05. **E**, **H** KM curves of OS for high- and low-risk subgroups in the training set and validation set. **F**, **I** ROC curves for the signature and its area under the curve (AUC) value represented 1-, 2-, and 3-year OS prediction in the training set (**F**) and validation set (**I**). **G**, **J** Risk score distribution plot (upper) showed the distribution of high-risk and low-risk LUAD patients in the training and validation set; Scatter plot (middle) showed the correlation between the survival status and risk score; Heatmap of six risk lncRNAs’ expression (lower) showed the expression distribution in the high-risk and low-risk subgroups

Through the co-expression analysis between the 2846 m^6^A-modified lncRNAs and 30 m^6^A regulators from the criteria of |Pearson.*r*| > 0.3 and *P* < 0.001 in TCGA-LUAD set, 215 lncRNAs were defined as m^6^A-regulated lncRNAs (Fig. 5A and Additional file 2: Table S4). Then, we recognized that 13 m^6^A-regulated lncRNAs were correlated with the OS of patients in the TCGA-LUAD set by univariate Cox regression analysis (*P* < 0.01) (Fig. 5B). The co-expressions of 13 m^6^A-regulated lncRNAs and m^6^A regulators were showed in Fig. 5C. The differences of clinical characteristics between the training and validation sets were ruled out (Table 1), implying that the random division of TCGA-LUAD samples had no effect on subsequent analysis. Next, LASSO Cox analysis and lambda value were used to eliminate 13 m^6^A-regulated lncRNAs that were highly correlated with each other to avoid overfitting in the training set (Fig. 6A, B). Finally, this produced a m^6^A-regulated lncRNA prognostic signature containing six lncRNAs and the coefficient of each (Fig. 6C). This used the following formula: risk score = 0.109 * AL590666.2 + (− 0.024) * CH17-340M24.3 + 0.291 * MIR31HG + (− 0.493) * MIR99AHG + (− 0.186) * LINC01936 + 0.111 * LINC02802. Then, RT-qPCR assays on LUAD clinical tissue samples were used to preliminarily explore the expression levels of the six lncRNAs in the signature. As shown in Fig. 6D, the expression of AL590666.2, a risk factor of OS (HR = 1.232, 95% CI: 1.104–1.375), was increased in LUAD tumor tissues (*P* < 0.05), while the expression of LINC01936, a protective factor (HR = 0.721, 95% CI: 0.567–0.917), was decreased in LUAD tumor tissues compared with corresponding normal tissues (*P* < 0.05). The expression levels of other prognostic m^6^A-regulated lncRNAs had no change (all *P* > 0.05).

**Table 1:** Comparison of clinical characteristics of TCGA-LUAD patients in the training and validation sets

Covariates	Type	Overall set (*N* = 500)	Training set (*N* = 252)	Validation set (*N* = 248)	*P* value
Age	> = 65	271 (54.2%)	137 (54.4%)	134 (54.0%)	0.802
	< 65	219 (43.8%)	111 (44.0%)	108 (43.5%)	
	Unknown	10 (2%)	4 (1.6%)	6 (2.4%)	
Gender	Female	270 (54%)	141 (56.0%)	129 (52.0%)	0.377
	Male	230 (46%)	111 (44.0%)	119 (48.0%)	
Stage	I–II	387 (77.4%)	200 (79.4%)	187 (75.4%)	0.258
	III–IV	105 (21%)	50 (19.8%)	55 (22.2%)	
	Unknown	8 (1.6%)	2 (0.8%)	6 (2.4%)	
T	1–2	434 (86.8%)	218 (86.5%)	216 (87.1%)	> 0.999
	3–4	63 (12.6%)	32 (12.7%)	31 (12.5%)	
	Unknown	3 (0.6%)	2 (0.8%)	1 (0.4%)	
N	0	324 (64.8%)	164 (65.1%)	160 (64.5%)	0.641
	1–3	165 (33%)	81 (32.1%)	84 (33.9%)	
	Unknown	11 (2.2%)	7 (2.8%)	4 (1.6%)	
M	0	332 (66.4%)	173 (68.7%)	159 (64.1%)	0.534
	1	24 (4.8%)	12 (4.8%)	12 (4.8%)	
	Unknown	144 (28.8%)	67 (26.6%)	77 (31.0%)

Subsequently, for evaluating the performance of the prognostic risk-related signature, the TCGA-LUAD patients were assigned to low- and high-risk subgroups according to the median value of risk scores of the training set. KM survival curves depicted that the survival probability of the high-risk subgroup was lower (*P* < 0.001 in training set; *P* = 0.005 in validation set) (Fig. 6E, H). ROC curves showed that the AUC values of the 1-, 2-, and 3-year OS prediction for the signature were all greater than 0.66 (Fig. 6F, I). In addition, the risk score distribution plots and scatter plots showed that the high-risk subgroup had shorter survival times than the low-risk subgroup (upper and middle parts of Fig. 6G, J). The cluster heatmaps showed expression differences of the six prognosis-related m^6^A-regulated lncRNAs between the two subgroups (lower part of Fig. 6G, J).

### Independence assessment of the signature and stratification analysis based on clinical features

**Fig. 7:**
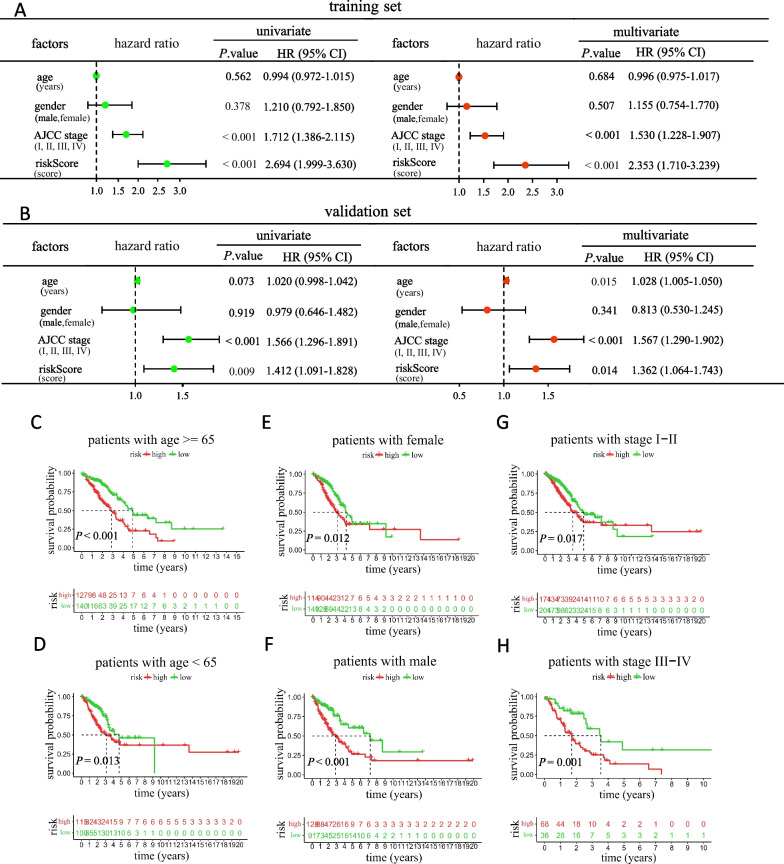
Independence assessment and stratification analyses of the prognostic signature based on clinicopathological features. **A**, **B** Forest plots of univariate and multivariate Cox regression analyses in the training set (**A**) and validation set (**B**). **C**-**H** KM curves showed the survival outcomes of high- and low-risk subgroups stratified by age (≥ 65 vs. < 65 years old) (**C**, **D**), gender (female vs. male) (**E**, **F**), and AJCC stage (I–II vs. III–IV) (**G**, **H**)

As revealed by univariate Cox regression analyses, risk score predicted undesirable OS (HR = 2.694, 95% CI: 1.999–3.630 in training set; HR = 1.412, 95% CI: 1.091–1.828 in validation set; and all *P* < 0.01) as well as AJCC stage (HR = 1.712, 95% CI: 1.386–2.115 in training set; HR = 1.566, 95% CI: 1.296–1.891 in validation set; and all *P* < 0.001) (left parts of Fig. 7A, B). Moreover, the multivariate analyses simultaneously confirmed the independence of our constructed risk score signature for predicting the prognosis of LUAD patients (HR = 2.353, 95% CI: 1.710–3.239 in training set; HR = 1.362, 95% CI: 1.064–1.743 in validation set; and all *P* < 0.05) as well as AJCC stage (HR = 1.530, 95% CI: 1.228–1.907 in training set; HR = 1.567, 95% CI: 1.290–1.902 in validation set; and all *P* < 0.001) (right parts of Fig. 7A, B).

We employed stratification analysis to confirm the prognostic value of the 6-MRlncRNA prognostic signature. The 482 TCGA-LUAD set patients with complete required clinical features were stratified into different groups based on age (≥ 65 and < 65 years old), gender (female and male) and tumor stage (I-II and III-IV), and were classified into high- and low-risk subgroups in each group according to the median risk score. As expected, the KM survival curves showed that the high-risk subgroup had worse OS compared with the low-risk subgroup in all strata of clinical characteristics (*P* < 0.05; Fig. C–H), suggesting that the risk score could accurately distinguish the prognosis of the populations with different characteristics. Overall, the stratification analyses indicated that the lncRNA prognostic signature was effective.

### Construction of the m^6^A-induced CeRNA network

**Fig. 8:**
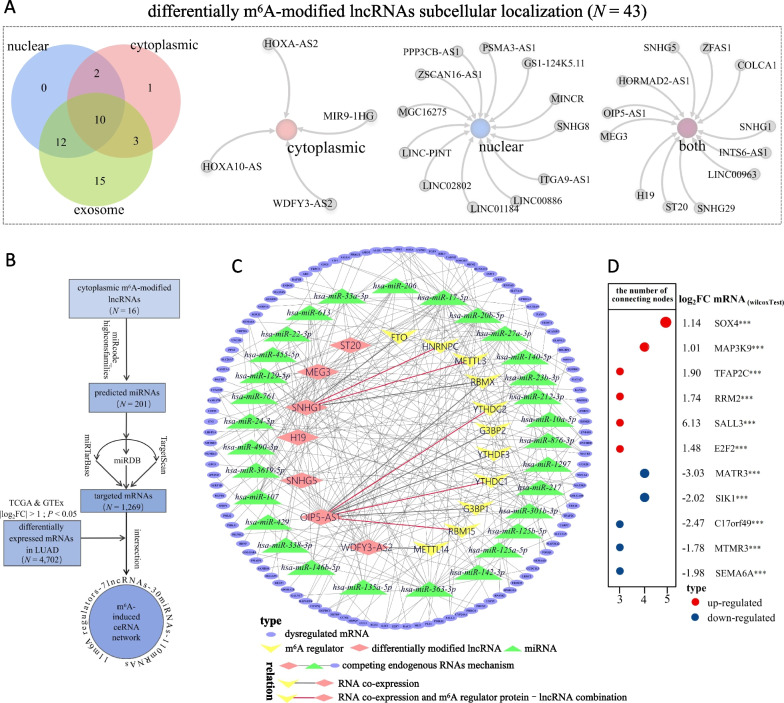
Construction of m^6^A-induced ceRNA Network. **A** The subcellular localization of 43 differentially m^6^A-modified lncRNAs according to RNALoate. **B** The construction process of the ceRNA network, which consisted of 11 correlative m^6^A regulators, seven differentially modified lncRNAs, 30 sponged miRNAs, and 110 targeted mRNAs. **C** The diagram of the ceRNA network. The lines connecting m^6^A regulators and lncRNAs represented RNA co-expression in TCGA-LUAD set (|*r*|> 0.3 and *P* < 0.001), and the red line represented the lncRNA-m^6^A regulator protein combination detected by CLIP-seq recorded in the POSTAR3 database. **D** The chart emphasized the dysregulated targeted mRNAs in LUAD, whose numbers of connecting nodes were greater than or equal to 3 in the ceRNA network. FC represented the expression fold change of T-median/N-median. WilcoxTest. ***P < 0.001

In the 43 recorded cellular localizations of 143 differentially m^6^A-modified lncRNAs based on RNALocate, 40 lncRNAs were detected in extracellular exosomes. Four lncRNAs were present only in the cytoplasm, and 12 were found only in the nucleus, and 12 were present in both the cytoplasm and nucleus, the names of which were separately showed in Fig. 8A. All in all, a total of 16 lncRNAs were in the cytoplasm. Next, we used miRcode to identify 201 highly conserved miRNAs interacting with 16 cytoplasmic lncRNAs. Subsequently, three databases combined to predict 1269 miRNAs-targeted mRNAs. Simultaneously, a total of 4702 differentially expressed mRNAs (|log_2_FC|> 1, *P* < 0.05) in LUAD were screened out by the combined analysis of TCGA and GTEx. Ultimately, after taking the intersection, 11 correlative m^6^A regulators, seven differentially m^6^A-modified lncRNAs, 30 sponged miRNAs, and 110 targeted mRNAs formed the ceRNA network (Fig. 8B, C). Furthermore, we listed 11 mRNAs with the number of connecting nodes greater than or equal to 3 in the regulatory network (Fig. 8D), which had a high probability to be regulated by the ceRNA network induced by differential m^6^A modification on lncRNA in LUAD. Simultaneously, we labeled the dysregulated expression levels of 11 mRNAs in LUAD tumor tissues, which had significant fold changes (|log_2_FC|> 1, *P* < 0.001) (Fig. 8D).

## Discussion

After studies on histone acetylation and DNA 5-methylcytosine modification, RNA m^6^A methylation has become another epigenetics focus that has attracted much attention in cancer research in recent years. The overall RNA m^6^A modification abundances in tumors were reportedly frequently abnormal. Ma et al. observed that the m^6^A% level of total RNA displayed a gradient descending trend in normal liver tissues, adjacent tissues, and tumor tissues of patients with hepatocellular carcinoma [22]. Ge et al. validated that the m^6^A% in total RNA of gastric tumor cells was significantly higher than that in normal gastric epithelial cells [23]. Here, we observed a decreased tendency of m^6^A modification abundance in LUAD tumor tissues at stage I and in most LUAD cell lines. These results indicated that m^6^A was vital for tumorigenesis in early-stage LUAD. Using statistical analysis and validation experiments, we found that multiple m^6^A regulators were abnormally expressed at both the RNA and protein levels in LUAD tissues, which may lead to the dysregulation of the m^6^A%. Furthermore, a majority of m^6^A regulators were related in expression patterns and functionally synergistic in LUAD, which were consistent with other reported tumor types [24, 25]. Overall, the m^6^A modification level was dysregulated and potentially involved in the occurrence and development of LUAD.

In recent years, studies have demonstrated that the dysregulation of m^6^A modifications on lncRNAs mediated by m^6^A regulators could change the lncRNA expression levels and further facilitate tumor malignant progression. For example, in lung cancer, the stability of MALAT1 was increased by its hyper m^6^A modification level induced by METTL3. Then, the upregulated MALAT1 could bind miR-1914-3p to further regulate the expression of YAP, a co-transcription factor upregulating chemo-resistant genes [26]. In gastric cancer, METTL14-mediated m^6^A modification led to LINC01320 upregulation, which promoted an aggressive phenotype of increased cancer cell proliferation, migration, and invasion via regulating the miR-495-5p/RAB19 axis [27]. However, relevant studies on the m^6^A-modified lncRNA profile in LUAD are still in a preliminary stage and are not fully comprehensive. Among the 2846 m^6^A-modified lncRNA transcripts detected in our epitranscriptomic microarray, we observed that the m^6^A-modified lncRNA with the highest proportion in LUAD tumor tissues had the following characteristics: exon sense-overlapping and intergenic transcript type, derived from chromosome 1 or 2, and was within the 500 to 1000 bp range. Among the 143 differentially m^6^A-modified lncRNA transcripts, about 55% concurrently associated with an altered expression level, which may be worthy of more in-depth studies of functional mechanisms. This phenomenon was confirmed by the experimental validation of PCAT19, SOCAR and SNHG8. According to the fold change analyses, we inferred that in LUAD, small variations of m^6^A modification levels on lncRNAs may cause a significant alteration of its expression levels. Additionally, we noted that in both normal and tumor tissues, differentially modified lncRNAs with higher m^6^A modification levels had lower expression levels. Overall, this study screened out specific m^6^A-modified lncRNAs and complemented existing research about the relationship between m^6^A modification and expression levels.

Accumulating evidence indicated that m^6^A modification was an important activator or stabilizer for lncRNAs [26, 28–30]. Therefore, we considered that the functionality of m^6^A-modified lncRNA may be stronger than that of its unmodified form, and was more likely to be an accurate and effective tumor biomarker. We identified the m^6^A-modified lncRNAs co-expressed with m^6^A regulators as the “m^6^A-regulated lncRNAs”, from which we constructed and verified a new prognostic signature that could successfully predict the OS of LUAD patients from their risk scores. The signature was subsequently demonstrated to be an independent prognostic factor, have favorable stability in different clinicopathological subgroups, and be a reliable metric to assess survival time of LUAD patients. Of the six m^6^A-regulated lncRNAs included in the signature, we verified that AL590666.2 was increased and LINC01936 was decreased in LUAD tumor tissues, while the expression levels of the other lncRNAs were not statistically different. AL590666.2 and CH17-340M24.3 had not been previously reported in tumor. MIR31HG participated in an immune-related lncRNA signature to predict survival and immunotherapy of LUAD patients [31]. In addition, MIR31HG targeted HIF1A and P21 to facilitate head and neck cancer cell proliferation and tumorigenesis by promoting cell cycle progression [32]. In pancreatic tumors, aberrantly upregulated MIR99AHG could modulate notch receptor 2 (NOTCH2) expression and stimulate the Notch signaling pathway to accelerate malignant progression through sponging miR-3129-5p and recruiting ELAV like RNA binding protein 1 (ELAVL1) [33]. In LUAD, LINC01936 displayed adequate performance in distinguishing LUAD patients from healthy people (AUC of ROC = 95.3%). Increased expression levels of LINC01936 were strongly related to a decreased risk of death [34]. LINC02802 was identified as a member of a prognostic signature for cervical tumors [35].

M^6^A modifications not only affected the lncRNA expression levels, but also directly modulated the ceRNA model of lncRNAs. A recent study demonstrated that decreased m^6^A modification level of LINC1281 attenuated its interaction with let7, suggesting that the m^6^A modification was necessary for the LINC1281-mediated ceRNA model [28]. Similarly, the ZFAS1-miR-647 interaction was regulated by METLL3-mediated m^6^A modification on ZFAS1 [29]. To explore the underlying mechanism, we constructed a ceRNA model originating from differentially m^6^A-modified lncRNAs localized in the cytoplasm, the axis of which included 11 m^6^A regulators, seven lncRNAs, 30 miRNAs, and 110 abnormally expressed mRNAs. 11 mRNAs, SOX4, MAP3K9, MATR3, TFAP2C, RRM2, SALL3, E2F2, C17orf49, MTMR3, SEMA6A and SIK1, were most likely modulated by hyper or hypo m^6^A modification levels on lncRNAs in LUAD tumor tissues. Interestingly, the subcellular localization results showed that most differentially m^6^A-modified lncRNAs in LUAD were observed in exosomes, a type of extracellular vesicles. In line with the hypothesis put forward by other researchers [36], m^6^A modifications may favor specific packaging of lncRNAs into exosomes. Given that m^6^A can regulate many RNA metabolic processes, including stability, translation, splicing, and structure switch [37], it would be meaningful to investigate if exosomes serve as intercellular transport carriers of tumor-associated m^6^A-modified RNAs in the transformation of target cells from normal to malignant.

The present study had some limitations. Although the microarray analysis revealed specific m^6^A-modified lncRNAs benefiting from specific hybridization probes, we could not determine the exact sites and motif sequences of m^6^A modification. Additionally, the overall m^6^A modification level of a full-length lncRNA may cover up the methylation difference at one specific site because multiple m^6^A sites were often on one lncRNA transcript. The reliability of the proposed prognostic signature needs to be further evaluated using external data. Furthermore, the m^6^A-induced ceRNA model requires rigorous molecular experiments to be validated. Even so, our study is meaningful and pioneering, as we have revealed the molecular features, prognostic values, and regulatory functionalities of m^6^A-modified lncRNAs in LUAD, providing references for the future research in this field.

## Methods

### Clinical tissue sample collection and cell culture

Twenty pairs of tumor and adjacent normal tissues were collected from LUAD patients undergoing surgical treatment in Shanghai Tongji Hospital from 2018 to 2021. The patients were screened to have no history of other malignancies and not receive any preoperative radiotherapy or chemotherapy. The clinicopathological characteristics of the study subjects were summarized in Additional file 2: Table S5. The study was approved by the hospital’s medical ethics committee with written informed consent received from all patients. H1792, H1975, H838, HCC827, and H1944 cell lines were cultured in RPMI-1640 medium (Hyclone, South Logan, UT, USA). A549, PC9, and BEAS-2B cell lines were cultured in DMEM medium (Hyclone, South Logan, UT, USA). HBE cells were cultured in Keratinocyte medium (ScienCell, San Diego, CA, USA). All medium contained 10% fetal bovine serum (FBS; Gibco, Grand Island, NY, USA) and all cells were incubated at 37 °C and 5% CO_2_.

### RNA extraction and RT-qPCR

Total RNA was extracted using Trizol reagent (Invitrogen, Carlsbad, CA, USA), and genomic DNA was removed using Recombinant DNase I (Takara, Dalian, China). 5 × Evo M-MLV RT Master Mix (AG, Changsha, China) was used for cDNA synthesis. SYBR^®^ Green Premix Pro Taq HS qPCR Kit II (AG, Changsha, China) was used for qPCRs, which were performed on a QuantStudio^®^ 5 real-time quantitative PCR instrument. The relative expression levels of targeted genes were calculated using the 2^(−∆∆CT)^ method using β-actin or GAPDH as the internal reference gene. The primers were listed in Additional file 2: Table S6.

### Quantification of total m^6^A RNA

The m^6^A content of approximate 200 ng RNA extracted from the indicated cells or tissues were analyzed using the EpiQuik m^6^A RNA Methylation Quantification Kit (Colorimetric) (Epigentek, Farmingdale, NY, USA) following the manufacturer’s instructions. The absorbance values were measured at a wavelength of 450 nm using a microplate reader within 15 min. The amount of m^6^A-modified RNA (ng) was calculated based on the standard curve. The value of m^6^A% was obtained by dividing the m^6^A amount by the input RNA amount.

### Western blot analysis and antibodies

Total protein was extracted using RIPA lysis buffer and was quantified using a BCA protein assay kit (Beyotime Biotechnology, Shanghai, China). Equal amounts of protein samples were separated by 10% SDS-PAGE and then transferred onto 0.45 μm nitrocellulose filter membranes (Cytiva, Amesham, UK). The membranes were blocked with 1 × protein free rapid blocking buffer (Epizyme Biotech, Shanghai, China) for 20 min at room temperature and then incubated with primary antibodies at 4 °C overnight. After three washes in Tris-buffered saline and Tween 20 (TBST), the membranes were incubated with horseradish peroxidase (HRP)-conjugated secondary antibodies (AP132P, 1:2000, Millipore, Billerica, MA, USA) for 1 h at room temperature. An ECL detection system (Tanon 4600SF, Shanghai, China) was used for visualization. β-Actin served as an internal control. The primary antibodies were as follows: β-actin (4970S, 1:1000, Cell Signaling Technology (CST), Beverly, MA, USA), METTL3 (86132S, 1:2000, CST, Beverly, MA, USA), METTL14 (26158-1-AP, 1:2000, Proteintech, Wuhan, China), ALKBH5 (80283S, 1:1000, CST, Beverly, MA, USA), FTO (27226-1-AP, 1:2000, Proteintech, Wuhan, China), IGF2BP1 (EPR26408-18, 1:2000, Abcam, Cambridge, UK), and IGF2BP3 (14642-1-AP, 1:2000, Proteintech, Wuhan, China).

### Acquisition of LUAD expression profiles and expression analysis of targeted genes

We downloaded the gene expression RNA-seq files from Genotype-Tissue Expression (GTEx) and TCGA, as well as their corresponding clinical phenotype and survival files from UCSC Xena (http://xena.ucsc.edu/). The sample GTEx-SUCS-0626-SM-5CHQE with abnormal value of gene expression was removed, and the data of 346 normal lung tissue samples and 526 LUAD tumor tissue samples were obtained. The gene set of two databases was intersected by the Perl program. The expression value of each gene was uniformly defined as log_2_FPKM+1. The normalize Between Arrays function of limma *R* package was used to correct the data. Subsequently, genes were categorized into lncRNA and mRNA groups using the human genome annotation file of GRCH38.p13 version. The Wilcox Test was used to analyze the differential expression of targeted genes.

### Correlation analysis and string protein interaction network of m^6^A regulators

Pearson correlation analysis was performed on the expression levels of 30 m^6^A regulators in 872 lung tissues. The Cor.mtest function was used to calculate the significance of correlation coefficients and *P* < 0.001 was considered to be statistically significant. The correlation heatmap was plotted by the “corrplot” *R* package. In addition, we analyzed the String protein interaction network (https://www.string-db.org/) of 30 m^6^A regulators with a confidence score cutoff greater than 0.6 as the screening condition. The active interaction sources included textmining, experiments, databases, co-expression, neighborhood, and co-occurrence. Cytoscape was utilized to visualize the protein–protein interaction (PPI) network.

### M^6^A-modified LncRNA epitranscriptomic microarray

We performed the Arraystar Human m^6^A-lncRNA Epitranscriptomic Microarray detection on six paired LUAD tumor tissues and adjacent normal tissues confirmed by pathological examination. The entire experimental procedure performed by Shanghai Aksomics Biotechnology Co., Ltd was shown in Additional file 1: Fig. S2. The m^6^A methylation levels and expression levels of lncRNA transcripts were calculated according to the following formulas:

$${\text{log}}_{2} \left( {{\text{IP}}_{{{\text{Cy}}5{\text{ normalized intensity}}}} } \right) =\, {\text{log}}_{2} \left( {{\text{IP}}_{{{\text{cy}}5{\text{ raw}}}} } \right) - {\text{Average}}\left[ {{\text{log}}_{2} \left( {{\text{IP}}_{{{\text{spike}} - {\text{in}}\_{\text{Cy}}5{\text{ raw}}}} } \right)} \right]$$

$${\text{log}}_{2} \left( {{\text{Sup}}_{{{\text{Cy}}3{\text{ normalized intensity}}}} } \right) =\, {\text{log}}_{2} \left( {{\text{Sup}}_{{{\text{cy}}3{\text{ raw}}}} } \right) - {\text{Average}}\left[ {{\text{log}}_{2} \left( {{\text{Sup}}_{{{\text{spike}} - {\text{in}}\_{\text{Cy}}3{\text{ raw}}}} } \right)} \right]$$

$${\text{\% Modified}} = \frac{{\text{Modified RNA}}}{{\text{Total RNA}}} =\, \frac{{{\text{IP}}}}{{{\text{IP}} + {\text{Sup}}}} =\, \frac{{{\text{IP}}_{{{\text{Cy}}5{\text{ normalized intensity}}}} }}{{{\text{IP}}_{{{\text{Cy}}5{\text{ normalized intensity}}}} + {\text{Sup}}_{{{\text{Cy}}3{\text{ normalized intensity}}}} }}$$

$${\text{Expression level of total RNA}} = {\text{IP}} + {\text{Sup}} = {\text{IP}}_{{{\text{Cy}}5{\text{ normalized intensity}}}} + {\text{Sup}}_{{{\text{Cy}}3{\text{ normalized intensity}}}}$$

### MeRIP-qPCR

Three µg total RNA was added to 60 µL 5 × IP buffer (50 mM Tris HCl pH 7.4, 750 mM NaCl, 0.5% IGEPAL CA-630) containing 2 µg affinity purified anti-m^6^A rabbit polyclonal antibody (Synaptic Systems, Goettingen, Germany), and then incubated in a rotary shaker for 2 h at 4 °C. A rabbit anti-human IgG antibody (Abcam, Cambridge, UK) was used as negative control. The samples were then mixed with 20 µL sheep anti-rabbit IgG magnetic beads (Invitrogen, Carlsbad, CA, USA) that were blocked in advance with 0.5% BSA for 2 h. The mixture was then incubated at 4 °C for 14 h. The beads were washed three times with 300 µL 1 × IP buffer and twice with 300 µL 1 × wash buffer (100 mM Tris HCl pH 7.4, 50 mM NaCl, 0.1% IGEPAL CA-630). The m^6^A-modified RNA was eluted from the magnetic beads using 300 µL elution buffer (100 mM Tris HCl pH 7.4, 1 mM EDTA, 0.05% SDS, 4 µL proteinase K, 2 µL RNase inhibitor) in the rotary shaker for 1 h at 50 °C. The RNA was extracted by phenol/chloroform/isoamylol (25:24:1) reagent (Tinadz, Beijing, China). The input RNA and m^6^A-modified RNA were both analyzed via RT-qPCR in a 1:1 ratio. The primers are listed in Additional file 2: Table S6. The m^6^A methylation level of lncRNA was calculated using the following formula:

$$\Delta {\text{CT}}_{{{\text{RIP}}}} = {\text{CT}}_{{\text{m6A - IP}}} {-}{\text{ CT}}_{{{\text{Input}}}}$$

$$\% {\text{Input}} = 2{\mkern 1mu}^{ \wedge } \left( { - \Delta {\text{CT}}_{{{\text{RIP}}}} } \right) \times 100\%$$

### Construction and validation of m^6^A-regulated LncRNA prognostic signature

According to previous publications, 30 proven m^6^A regulators were included, including methyltransferases, demethylases, and binding functional proteins. Through the Pearson correlation analysis (|*r*|> 0.3 and *P* < 0.001) of expression level, a total of 2017 lncRNAs related to m^6^A regulators were verified, including 215 m^6^A-modified lncRNAs screened by m^6^A microarray. Samples without survival values were removed out, leaving 500 TCGA-LUAD tumor samples to be included in the subsequent survival analysis. Next, 500 samples were randomly divided into a training set (*N* = 252) and validation set (*N* = 248) for the construction and validation of the prognostic signature, respectively. Later, the least absolute shrinkage and selection operator (LASSO) regression analysis was applied to eliminate those prognostic-associated lncRNAs highly correlated with each other to avoid overfitting. The risk score of LUAD patients was calculated using the following formula:

$${\text{Risk}}\;{\text{score}} = \sum\limits_{i = 1}^{n} {{\text{coef}}\left( i \right) * {\text{lncRNA}}\left( i \right)_{{{\text{expression}}}} }$$

The LUAD patients were classified into high-risk and low-risk subgroups using the median risk score as the cutoff value. Kaplan–Meier (KM) survival curve analysis was performed to compare the survival outcomes of the two subgroups. The receiver operating characteristic curve (ROC) and its area under the curve (AUC) values were utilized to evaluate the specificity and sensitivity of the signature using the “timeROC” *R* package. The information and risk score for each sample in the training and validation set were supplemented in the Additional file 2: Table S7.

### Independence assessment of the signature and stratification analysis

We excluded the TCGA-LUAD tumor samples with unknown clinical variables of age, gender and AJCC stage. A total of 482 tumor samples (Additional file 2: Table S7) were included to evaluate the independence of the signature using univariate and multivariate Cox regression analyses, and to assess the signature’s ability to predict OS in different groups of age (≥ 65 and < 65 years old), gender (female and male) and AJCC tumor stage (I-II and III-IV).

### Construction of m^6^A-induced competitive endogenous RNA (CeRNA) network

To clarify the competitive endogenous regulation of differentially m^6^A-modified lncRNAs, we firstly analyzed their cellular localization using the RNALocate v2.0 database (http://www.rna-society.org/rnalocate/). Next, miRcode (http://www.mircode.org/) was used to identify highly conserved miRNAs interacting with lncRNAs. Subsequently, miRNAs-targeted mRNAs were predicted by three databases, which were TargetScan (http://www.targetscan.org/), miRDB (http://www.mirdb.org/), and miRTarBase (http://mirtarbase.mbc.nctu.edu.tw). Only mRNAs predicted by all three databases were eligible. Simultaneously, differentially expressed mRNAs in LUAD were screened out with the combined analysis of TCGA and GTEx. The combination of lncRNA–m^6^A regulator protein detected by CLIP-seq was recorded in the POSTAR3 database (http://111.198.139.65/RBP.html). Cytoscape was utilized to visualize the ceRNA regulatory relationship.

## Supplementary Information

**Additional file 1**. Additional figures S1–2.

**Additional file 2**. Additional Tables S1–7; microarray row data; normalized m^6^A lncRNA microarray.

## Data Availability

All data in the current study are available from the corresponding author on reasonable request.

## Abbreviations

LUAD: Lung adenocarcinoma
m^6^A: N6-methyladenosine
lncRNAs: Long non-coding RNAs
ROC: Receiver operating characteristic curve
AUC: Area under the curve
TCGA: The Cancer Genome Atlas
GTEx: Genotype-Tissue Expression
OS: Overall survival
AJCC: American Joint Committee on Cancer
FDR: False discovery rate
ceRNA: Competitive endogenous RNA
