# Supplementary material for: Comprehensive analyses of molecular features, prognostic values, and regulatory functionalities of m6A-modified long non-coding RNAs in lung adenocarcinoma
Source: Clin Epigenetics. 2023 Apr 7;15:60. doi: 10.1186/s13148-023-01475-z (PMC10082542; doi:10.1186/s13148-023-01475-z)
Supplement: Supplementary file 1 — Additional file 1. Additional figures S1–2. [file 13148_2023_1475_MOESM1_ESM.pdf]

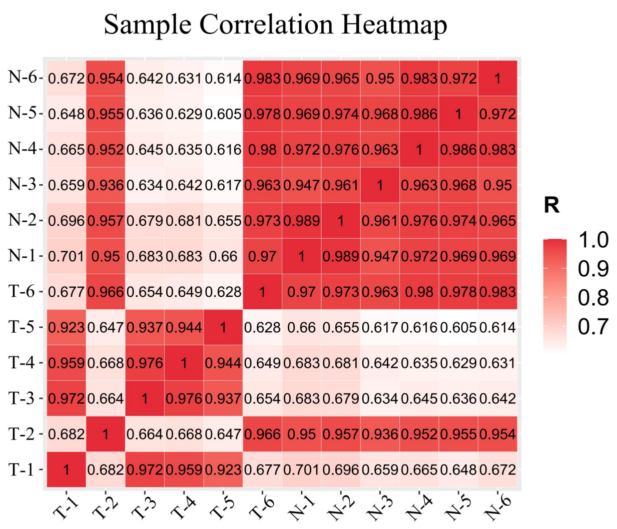

Fig. S1 The sample correlation analysis of m^{6}A-modified lncRNA microarray based on the lncRNAs' expression levels in six paired tumor tissues (T1-6) and adjacent normal tissues (N1-6).

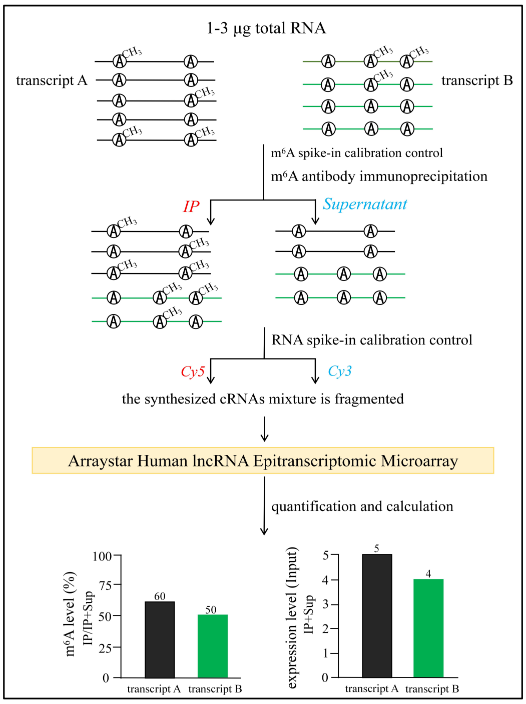

Fig. S2 The experimental process diagram of m^{6}A-modified lncRNA epitranscriptomic microarray. Briefly, the total RNAs were immunoprecipitated with anti-N6-methyadenosine antibody. The modified RNAs were eluted from the immunoprecipitated magnetic beads as the "IP". The unmodified RNAs were recovered from the supernatant as "Sup". The "IP" and "Sup" RNAs were labeled with Cy5 and Cy3 respectively as cRNAs in separate reactions using Arraystar Super RNA Labeling Kit. The cRNAs were combined together and hybridized onto Arraystar Human lncRNA Epitranscriptomic Microarray (8x60K, Arraystar). After washing the slides, the arrays were scanned in two-color channels by an Agilent Scanner G2505C. Agilent Feature Extraction software (version 11.0.1.1) was used to analyze acquired array images.
